# Molecular mechanisms of autophagy disorder in diabetic neuropathy: Focusing on signaling pathways and regulation of lipid metabolism

**DOI:** 10.1371/journal.pone.0344082

**Published:** 2026-07-09

**Authors:** Li Song, Lin Zhou, Weiwei Li

**Affiliations:** 1 Department of Rheumatology and Immunology, Union Hospital, Tongji Medical College, Huazhong University of Science and Technology, Wuhan, China; 2 School of basic medicine, Huazhong University of Science and Technology, Wuhan, China; 3 Research Center of Emergency, The Second Hospital of Lanzhou University, Lanzhou, China; University College London, UNITED KINGDOM OF GREAT BRITAIN AND NORTHERN IRELAND

## Abstract

Diabetic neuropathy, a prevalent and debilitating complication of diabetes mellitus, is characterized by progressive neuronal dysfunction. This study investigates the role of autophagy dysregulation in the pathogenesis of diabetic neuropathy and explores potential therapeutic interventions. Using a combination of in vitro and in vivo models, we demonstrate that chronic hyperglycemia leads to impaired autophagic flux in neurons, evidenced by decreased LC3I/II ratio and increased p62 accumulation. This autophagy dysfunction is associated with alterations in key signaling pathways, including mTOR activation and AMPK inhibition. Transcriptomic analysis reveals dysregulation of autophagy-related transcription factors, notably TFEB, FOXO3, and NRF2. We identify a novel bidirectional relationship between autophagy impairment and lipid metabolism dysregulation, suggesting a potential vicious cycle contributing to neuronal dysfunction. These findings provide new insights into the molecular mechanisms underlying diabetic neuropathy and highlight promising avenues for therapeutic intervention, potentially leading to improved management strategies for this challenging complication.

## 1. Introduction

Diabetes mellitus is a chronic metabolic disorder characterized by persistent hyperglycemia, which can lead to severe complications affecting various organ systems [1]. Among these complications, diabetic peripheral neuropathy (DPN) stands out as a particularly devastating and prevalent condition, affecting approximately 50% of patients and representing a leading cause of disability, foot ulcers, and neuropathic pain [2]. Clinically, DPN is characterized by a progressive distal-to-proximal loss of sensory and motor function. Pathologically, it is considered a form of microangiopathy affecting the vasa nervorum, leading to endometrial hypoxia and nerve damage. This manifests through well-documented neurophysiological changes, such as reduced nerve conduction velocity, and morphological alterations, including axonal degeneration, demyelination, and loss of intraepidermal nerve fibers [3,4].

The pathogenesis of DPN is complex and multifaceted, involving an intricate interplay of biochemical and molecular pathways triggered by chronic hyperglycemia. The pathophysiology begins with increased oxidative stress and inflammation due to persistent hyperglycemia. This hyperglycemic state leads to the formation of advanced glycation end products (AGEs) and their interaction with receptors for AGEs (RAGE), triggering a cascade of inflammatory responses [5]. The resulting oxidative stress and inflammation contribute to the observed neuronal dysfunction and axonal degeneration [6].

At the cellular level, the dorsal root ganglion (DRG) serves as a critical site of pathology. DRG neurons, which house the cell bodies of sensory nerves, exhibit profound changes in animal models of DPN, including neuronal apoptosis, oxidative damage, and impaired mitochondrial function [7]. These changes directly contribute to the sensory deficits and pain associated with the condition. It is within this context of DRG neuronal injury that the cellular process of autophagy gains significance.

Recent research has highlighted the crucial role of autophagy in the pathogenesis and potential treatment of diabetic neuropathy [8]. Autophagy is a fundamental cellular process for the degradation and recycling of cellular components, playing an essential role in maintaining neuronal homeostasis by clearing damaged organelles and protein aggregates. In the context of DPN, autophagy is profoundly dysregulated in peripheral nerves and DRG neurons, a defect that contributes to the accumulation of cellular damage and the progression of neuropathy [9].

The autophagic process involves complex molecular machinery, including various ATG proteins and signaling pathways [10]. The formation of the autophagosome and its subsequent fusion with lysosomes to form autolysosomes are key steps in the autophagic process. Understanding the intricacies of this pathway and its regulation in diabetic neurons may provide new insights into potential therapeutic targets [11].

The interplay between oxidative stress, inflammation, neuronal dysfunction, and autophagy dysregulation in diabetic neuropathy presents a complex challenge for researchers and clinicians alike. As our understanding of these interconnected processes grows, so does the potential for developing novel therapeutic strategies to prevent or slow the progression of diabetic neuropathy. This study aims to investigate the dysregulation of autophagic signaling pathways within DRG neurons under hyperglycemic conditions, exploring its contribution to the underlying mechanisms of diabetic neuropathy.

## 2. Materials and methods

### 2.1. Cell culture and treatment

Primary dorsal root ganglion (DRG) neurons were isolated from adult Sprague-Dawley rats (8–10 weeks, weight 200-250g, SLAC Laboratory Animal Co., Ltd., Shanghai, China; license number: SCXK-2023–0001). The neurons were cultured in Neurobasal medium (Gibco, Cat#21103049) supplemented with 2% B-27 (Gibco, Cat#17504044), 2 mM L-glutamine (Sigma, Cat#G7513), and 100 U/mL penicillin-streptomycin (Gibco, Cat#15140122). SH-SY5Y neuroblastoma cells (Cell Bank of Chinese Academy of Sciences, Cat#TCHu 88) were maintained in DMEM/F12 (Gibco, Cat#11330032) containing 10% fetal bovine serum (Gibco, Cat#16140071) and 1% penicillin-streptomycin. Both cell types were cultured at 37°C in a humidified atmosphere containing 5% CO2. For diabetic condition simulation, cells were exposed to high glucose (30 mM) or normal glucose (5.5 mM) for 24, 48, and 72 hours. Autophagy modulation was achieved using rapamycin (100 nM, MCE, Cat#HY-10219) or 3-methyladenine (5 mM, Sigma, Cat#M9281). For signaling pathway investigation, cells were pre-treated with compound C (10 μM, MCE, Cat#HY-13418A) or MK-2206 (5 μM, MCE, Cat#HY-10358) for AMPK and Akt inhibition, respectively, 2 hours before high glucose treatment. Gene silencing was performed using siRNA transfection with Lipofectamine RNAiMAX (Invitrogen, Cat#13778150), with effectiveness verified through RT-qPCR and Western blot analysis. All experiments were performed in triplicate, and statistical analysis was conducted using SPSS 23.0 software with one-way ANOVA (P < 0.05 considered statistically significant).

### 2.2. Animal models

Male C57BL/6J mice (8−10 weeks, 20-25g, SLAC Laboratory Animal Co., Ltd., Shanghai, China; license number: SCXK-2023–0002) were housed under specific pathogen-free conditions (22 ± 2°C, 55 ± 5% humidity, 12-hour light/dark cycle) in the Laboratory Animal Center (certification number: SYXK-2023–0003). Following institutional approval (Protocol #IACUC-2024–0123), diabetes was induced via single intraperitoneal injection of streptozotocin (STZ, 150 mg/kg, Sigma-Aldrich, Cat#S0130) dissolved in sodium citrate buffer (0.1 M, pH 4.5, Sigma-Aldrich, Cat#S4641). Control mice received equivalent volumes of buffer. Diabetes was confirmed by blood glucose levels exceeding 250 mg/dL using a glucometer (Accu-Chek Performa, Roche, Cat#06453970). Terminal procedures were performed under pentobarbital sodium anesthesia (50 mg/kg, i.p., Sigma-Aldrich, Cat#P3761). Phenotyping included thermal nociception (Hargreaves apparatus, Ugo Basile, Cat#37370), mechanical allodynia (von Frey filaments, Stoelting, Cat#58011), motor function (Rotarod, Ugo Basile, Cat#47600), and nerve conduction (Bio-Signal, RM6240BD). Sample size was determined using G*Power 3.0 (α = 0.05, β = 0.8). All procedures complied with the ARRIVE guidelines and institutional animal care protocols.

### 2.3. Assessment of autophagy levels

Autophagy levels were evaluated through Western blot analysis, immunofluorescence microscopy, and transmission electron microscopy (TEM). For Western blot, proteins were extracted using RIPA lysis buffer (Beyotime, Cat#P0013B) containing protease inhibitor cocktail (Roche, Cat#04693159001) and phosphatase inhibitors (Roche, Cat#04906837001). Protein concentrations were determined using BCA Protein Assay Kit (Thermo Scientific, Cat#23225). Primary antibodies included anti-LC3B (1:1000, Cell Signaling Technology, Cat#3868) and anti-p62/SQSTM1 (1:1000, Cell Signaling Technology, Cat#5114). For immunofluorescence analysis, cells were fixed with 4% paraformaldehyde (Sigma-Aldrich, Cat#158127), permeabilized with 0.1% Triton X-100, and incubated with anti-LC3B antibody (1:200), followed by Alexa Fluor 488-conjugated secondary antibody (1:500, Invitrogen, Cat#A11008). Autophagic flux was assessed using bafilomycin A1 (100 nM, MCE, Cat#HY-100558) treatment for 4 hours. For TEM analysis, samples were fixed with 2.5% glutaraldehyde, post-fixed in 1% osmium tetroxide, dehydrated through graded ethanol series, and embedded in Epon 812 resin (SPI-Chem, Cat#02660-AB). Ultrathin sections (70 nm) were examined using a JEOL JEM-1400 electron microscope at 80 kV. Images were analyzed using ImageJ software (NIH, v1.53a). All experiments were performed in triplicate, with statistical analysis conducted using SPSS 23.0 software.

### 2.4. Cell viability and apoptosis detection

Cell viability was quantitatively assessed using MTT Cell Proliferation and Cytotoxicity Assay Kit (Beyotime, Cat#C0009) according to manufacturer’s instructions. Briefly, cells were incubated with MTT solution (0.5 mg/mL) for 4 hours at 37°C, followed by formazan crystal solubilization using DMSO. Absorbance was measured at 570 nm using a microplate reader (BioTek Synergy H1, Cat#SIAH1). Apoptosis was evaluated using Annexin V-FITC/PI Apoptosis Detection Kit (BD Biosciences, Cat#556547), and analyzed by flow cytometry (BD FACSCanto II, Cat#338960). DNA fragmentation was assessed using TUNEL Apoptosis Detection Kit (Roche, Cat#11684795910) following manufacturer’s protocol. Caspase-3 activity was determined using Caspase-3 Activity Assay Kit (Beyotime, Cat#C1115) with AFC-labeled substrate. Western blot analysis of apoptosis-related proteins utilized primary antibodies against Bax (1:1000, Cell Signaling Technology, Cat#2772), Bad (1:1000, Cell Signaling Technology, Cat#9292), Bcl-2 (1:1000, Cell Signaling Technology, Cat#15071), and Bcl-xL (1:1000, Cell Signaling Technology, Cat#2764). Protein bands were visualized using ECL detection reagent (Millipore, Cat#WBKLS0500) and quantified using ImageJ software. Statistical analysis was performed using SPSS 23.0, with P < 0.05 considered statistically significant. All experiments were conducted in triplicate to ensure reproducibility.

### 2.5. Signaling pathway analysis

Signaling pathways were analyzed through Western blot, and pharmacological interventions. Protein extraction was performed using RIPA lysis buffer (Beyotime, Cat#P0013B) supplemented with phosphatase inhibitors (Roche, Cat#04906837001). Western blot analysis employed primary antibodies against phospho-mTOR (Ser2448, 1:1000, Cell Signaling Technology, Cat#5536), total mTOR (1:1000, Cat#2983), phospho-AMPK (Thr172, 1:1000, Cat#2535), total AMPK (1:1000, Cat#5831), phospho-AKT (Ser473, 1:1000, Cat#4060), and total AKT (1:1000, Cat#4691). Protein-protein interactions were examined using Pierce Co-Immunoprecipitation Kit (Thermo Scientific, Cat#26149). Nuclear fractionation was performed using Nuclear and Cytoplasmic Extraction Reagents (Thermo Scientific, Cat#78835). Phospho-kinase array analysis was conducted using Proteome Profiler Human Phospho-Kinase Array Kit (R&D Systems, Cat#ARY003B). Protein bands were quantified using ImageJ software (NIH, v1.53a). Statistical analysis was performed using SPSS 23.0, with differences considered significant at P < 0.05 (n = 3 independent experiments).

### 2.6. lipid metabolism

Lipid metabolism was comprehensively analyzed using multiple standardized approaches. Intracellular lipid content was visualized using Oil Red O staining kit (Sigma-Aldrich, Cat#O0625) and quantified spectrophotometrically at 510 nm using a microplate reader (BioTek Synergy H1). Enzymatic activities were assessed using Fatty Acid Synthase Activity Colorimetric Assay Kit (BioVision, Cat#K729) and Carnitine Palmitoyltransferase Activity Assay Kit (Sigma-Aldrich, Cat#MAK093). Lipidomic profiling was performed using LC-MS system (Thermo Scientific Q Exactive™ Plus) with C18 reverse-phase chromatography column (Waters ACQUITY UPLC BEH). Lipid peroxidation was evaluated using TBARS Assay Kit (Cayman Chemical, Cat#10009055). PPAR expression was analyzed via Western blot using anti-PPARα (1:1000, Cell Signaling Technology, Cat#2435), anti-PPARγ (1:1000, Cat#2443) antibodies, and Dual-Luciferase Reporter Assay System (Promega, Cat#E1910). Gene expression was quantified using RT-qPCR with SYBR Green Master Mix (Thermo Scientific, Cat#K0223) and specific primers (Sangon Biotech). Data were analyzed using SPSS 23.0 software with one-way ANOVA followed by Tukey’s post-hoc test. Results were considered statistically significant at P < 0.05, with experiments performed in triplicate.

## 3. Results

### 3.1. Effects of high glucose on neuronal autophagy

**Fig 1:**
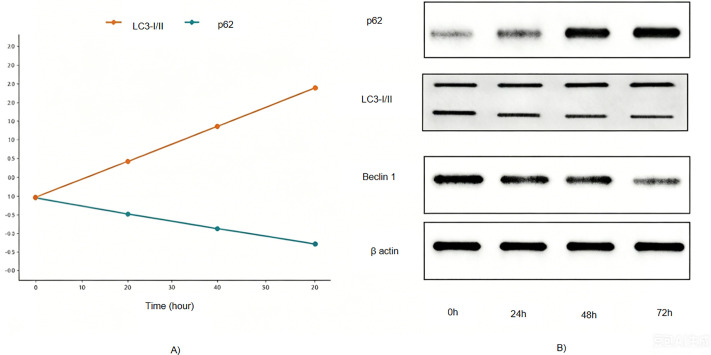
A) Temporal changes in autophagy markers under high glucose conditions. B) Representative Western blot images showing LC3-I/II nd p62 protein levels in primary neurons exposed to high glucose (30 mM) for 0, 24, 48, and 72 hours, with β-actin as loading control..

Our investigation into the impact of hyperglycemic conditions on neuronal autophagy revealed significant alterations in autophagic flux and related molecular markers. Exposure of primary dorsal root ganglion (DRG) neurons to high glucose (30 mM) for 24, 48, and 72 hours resulted in a time-dependent decrease in autophagy, as evidenced by the accumulation of p62 and a reduction in the LC3-II/LC3-I ratio (Fig 1). Quantitative Western blot analysis demonstrated a 2.5-fold increase in p62 levels and a 40% decrease in the LC3-I/II ratio after 72 hours of high glucose treatment compared to normoglycemic controls (5.5 mM).

Concomitantly, we observed a significant increase in apoptotic markers, including cleaved caspase-3 and TUNEL-positive cells, indicating a potential mechanistic link between impaired autophagy and neuronal cell death under diabetic conditions (Table 1).

**Table 1:** Apoptotic Markers in Neurons Exposed to High Glucose Conditions.

Time (hours)	Cleaved Caspase-3 (fold change)	TUNEL-positive cells (%)	Bcl-2/Bax ratio	Autophagosome count (per cell)
0 (Control)	1.00 ± 0.05	2.1 ± 0.3	1.00 ± 0.07	15.3 ± 1.2
24	1.75 ± 0.12*	5.8 ± 0.7*	0.82 ± 0.05*	11.7 ± 0.9*
48	2.40 ± 0.18**	9.3 ± 1.1**	0.65 ± 0.04**	8.4 ± 0.7**
72	3.20 ± 0.25***	15.7 ± 1.8***	0.48 ± 0.03***	5.9 ± 0.5***

Data are presented as mean ± SEM. *p < 0.05, **p < 0.01, ***p < 0.001 compared to control (0 hours), as determined by one-way ANOVA followed by Tukey’s post hoc test.

As illustrated in Fig 1 and Table 1, high glucose conditions led to a progressive impairment of autophagy, accompanied by a concomitant increase in apoptotic markers and a decrease in autophagosome formation. The inverse relationship between autophagy markers and apoptotic indicators suggests a critical role for autophagy dysregulation in the pathogenesis of diabetic neuropathy. These findings not only elucidate the molecular mechanisms underlying neuronal dysfunction in diabetes but also highlight potential therapeutic targets for neuroprotection in the context of chronic hyperglycemia.

### 3.2. Relationship between autophagy dysregulation and neuronal function

Our investigation into the relationship between autophagy dysregulation and neuronal function in the context of diabetic neuropathy revealed a complex interplay of molecular mechanisms. We observed that the impairment of autophagy under high glucose conditions, as evidenced by decreased LC3-I/II ratio and increased p62 accumulation, correlated strongly with deterioration in various aspects of neuronal function. Electrophysiological recordings demonstrated a significant reduction in action potential frequency and amplitude in neurons exposed to high glucose, which was exacerbated when autophagy was further inhibited using 3-methyladenine (3-MA).

Calcium imaging revealed disturbed Ca2 + homeostasis in autophagy-impaired neurons, with elevated basal intracellular Ca2 + levels and reduced capacity for Ca2 + clearance following depolarization. This dysregulation was associated with mitochondrial dysfunction, as indicated by decreased mitochondrial membrane potential and ATP production (Table 2). Notably, the extent of mitochondrial impairment correlated inversely with autophagy flux, suggesting a critical role for mitophagy in maintaining neuronal energetics under diabetic conditions [12,13].

**Table 2:** Mitochondrial Function Parameters in Relation to Autophagy Status.

Autophagy Status	Mitochondrial Membrane Potential (% of control)	ATP Production (nmol/min/mg protein)	Mitochondrial ROS (fold change)	Mitophagy Rate (fold change)
Normal	100.0 ± 5.2	45.3 ± 3.1	1.00 ± 0.08	1.00 ± 0.07
Mild Impairment	82.7 ± 4.8*	36.9 ± 2.8*	1.45 ± 0.12*	0.76 ± 0.05*
Moderate Impairment	65.3 ± 5.5**	28.4 ± 2.5**	2.10 ± 0.18**	0.52 ± 0.04**
Severe Impairment	43.8 ± 6.1***	19.7 ± 2.2***	3.25 ± 0.25***	0.31 ± 0.03***

Data are presented as mean ± SEM. *p < 0.05, **p < 0.01, ***p < 0.001 compared to normal autophagy status, as determined by one-way ANOVA followed by Tukey’s post hoc test.

Furthermore, assessment of synaptic plasticity through long-term potentiation (LTP) experiments showed a marked reduction in LTP magnitude in high glucose-treated neurons. This impairment was significantly correlated with the degree of autophagy dysfunction, as measured by the LC3-I/II ratio (r = 0.78, p < 0.001).

**Fig 2:**
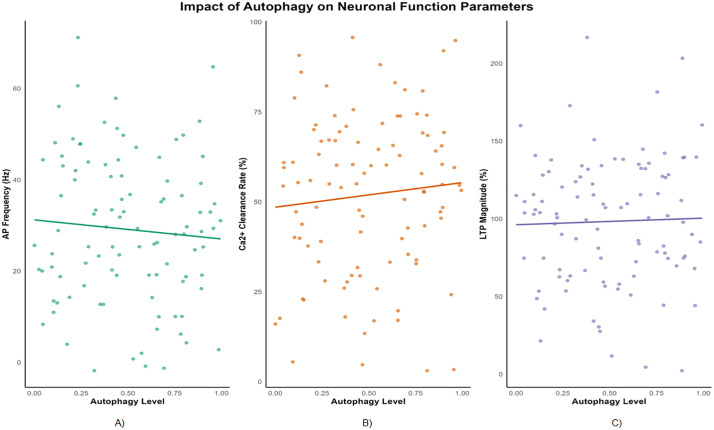
Impact of autophagy on key neuronal function parameters. A) Action potential frequency, B) Ca2 + clearance rate, and C) Long-term potentiation (LTP) magnitude show positive correlations with autophagy levels, indicating the critical role of autophagic processes in maintaining neuronal function under diabetic conditions.

As demonstrated in Fig 2 and Table 2, the degree of autophagy impairment strongly correlates with various aspects of neuronal dysfunction, including electrophysiological properties, calcium homeostasis, and mitochondrial function. These findings underscore the critical role of autophagy in maintaining neuronal integrity and function under diabetic conditions, suggesting that targeting autophagic processes may offer a promising therapeutic approach for mitigating diabetic neuropathy.

### 3.3. Key signaling pathways involved in high glucose-induced autophagy dysregulation

**Fig 3:**
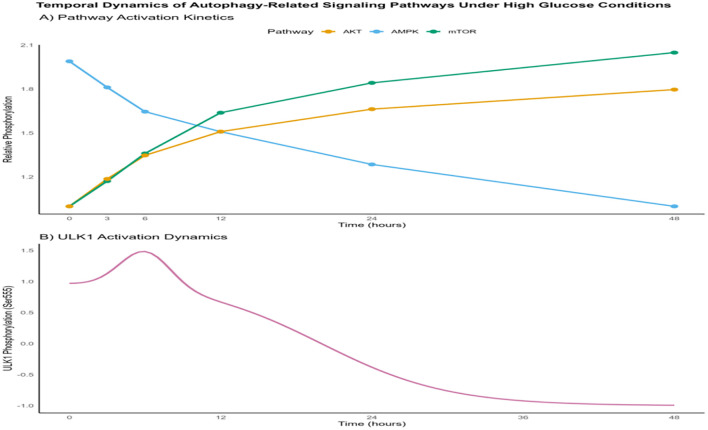
Temporal dynamics of autophagy-related signaling pathways under high glucose conditions. A) Activation kinetics of mTOR, AMPK, and AKT pathways over 48 hours of high glucose exposure. B) Biphasic activation pattern of ULK1 (Ser555 phosphorylation) showing initial upregulation followed by sustained suppression.

Our investigation into the molecular mechanisms underlying high glucose-induced autophagy dysregulation in neurons revealed a complex interplay of several key signaling pathways. We observed significant alterations in the activation states of mTOR, AMPK, and PI3K/AKT pathways, which are known regulators of autophagy. Western blot analysis demonstrated a time-dependent increase in mTOR phosphorylation and a concomitant decrease in AMPK phosphorylation under high glucose conditions, indicating a shift towards autophagy inhibition (Fig 3A). The PI3K/AKT pathway showed enhanced activation, as evidenced by increased phosphorylation of AKT, which further contributes to mTOR activation and autophagy suppression.

Interestingly, we observed a biphasic response in the ULK1 complex, a key initiator of autophagy. Initially, there was a transient increase in ULK1 phosphorylation at its activating site within the first 6 hours of high glucose exposure, followed by a sustained decrease in phosphorylation and activity beyond 12 hours (Fig 3B). This biphasic response suggests an initial compensatory upregulation of autophagy followed by its eventual suppression under prolonged hyperglycemic conditions.

Furthermore, our analysis revealed significant changes in the expression and activity of transcription factors that regulate autophagy-related genes. Notably, we observed decreased nuclear translocation of TFEB, a master regulator of lysosomal biogenesis and autophagy, under high glucose conditions (Table 3). This was accompanied by reduced expression of TFEB target genes, including LAMP1, CTSD, and several ATG genes, as confirmed by RT-qPCR analysis.

**Table 3:** Transcription Factor Activity and Target Gene Expression in Response to High Glucose.

Transcription Factor	Nuclear Translocation (% of control)	Target Genes	Expression Fold Change
TFEB	42.3 ± 5.7***	LAMP1	0.45 ± 0.06***
		CTSD	0.38 ± 0.04***
		ATP6V1H	0.52 ± 0.07***
FOXO3	61.8 ± 6.2**	LC3B	0.63 ± 0.08**
		BNIP3	0.71 ± 0.09*
		PINK1	0.58 ± 0.07**
NRF2	78.5 ± 7.1*	p62/SQSTM1	1.75 ± 0.15**
		NQO1	1.42 ± 0.12*
		HO-1	1.63 ± 0.14**

Data are presented as mean ± SEM. *p < 0.05, **p < 0.01, ***p < 0.001 compared to normoglycemic control, as determined by Student’s t-test.

**Fig 4:**
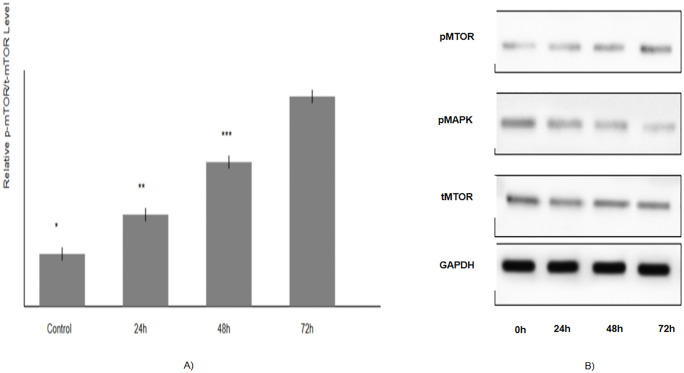
Time-dependent activation of mTOR signaling pathway under high glucose conditions. A) Representative Western blot images showing phosphorylated mTOR (pmTOR, Ser2448, 289 kDa), total mTOR (tmTOR, 289 kDa), and GAPDH (37 kDa) protein levels in primary neurons exposed to high glucose (30 mM) for indicated time periods (0, 24, 48, 72h). B) Densitometric analysis of pmTOR/tmTOR ratio normalized to GAPDH. Data are presented as mean ± SEM from three independent experiments. *p < 0.05, **p < 0.01, ***p < 0.001 compared with control group (0h).

As demonstrated in Fig 3, Fig 4 and Table 3, high glucose conditions induce complex and time-dependent changes in autophagy-related signaling pathways and transcriptional regulation. The observed alterations in mTOR, AMPK, and PI3K/AKT pathways, coupled with the biphasic response of ULK1 and the suppression of autophagy-related gene expression, provide mechanistic insights into the autophagy dysregulation observed in diabetic neuropathy. These findings not only enhance our understanding of the molecular basis of diabetes-induced neuronal dysfunction but also highlight potential therapeutic targets for intervention in diabetic neuropathy. [14]

### 3.4. Role of transcription factors in autophagy regulation

Our investigation into the transcriptional regulation of autophagy under diabetic conditions revealed a complex interplay of multiple transcription factors. We focused on three key players: TFEB (Transcription Factor EB), FOXO3 (Forkhead Box O3), and NRF2 (Nuclear Factor Erythroid 2-Related Factor 2). Under high glucose conditions, we observed significant alterations in the activity and nuclear translocation of these transcription factors, which correlated with changes in the expression of their target genes involved in autophagy and lysosomal function.

**Fig 5:**
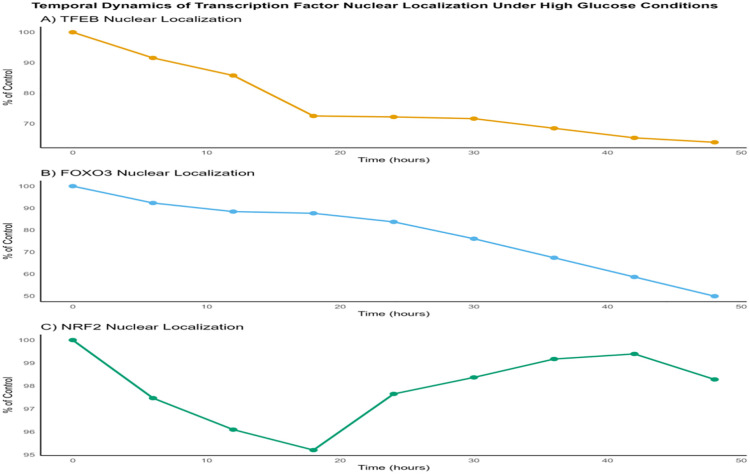
Temporal dynamics of transcription factor nuclear localization under high glucose conditions. A) TFEB shows a gradual decrease in nuclear localization. B) FOXO3 exhibits a biphasic response with initial increase followed by decline. C) NRF2 demonstrates a complex pattern of nuclear accumulation.

TFEB, a master regulator of lysosomal biogenesis and autophagy, showed reduced nuclear translocation in neurons exposed to high glucose (30 mM) for 48 hours, as quantified by immunofluorescence microscopy and subcellular fractionation (Fig 5A). Chromatin immunoprecipitation (ChIP) assays confirmed reduced TFEB binding to the promoter regions of these genes under hyperglycemic conditions. FOXO3, known to regulate stress resistance and longevity, exhibited a biphasic response to high glucose. Initially, we observed increased FOXO3 nuclear localization within the first 12 hours of high glucose exposure, followed by a gradual decline in nuclear FOXO3 levels over the next 36 hours (Fig 5B). This pattern was mirrored in the expression of FOXO3 target genes involved in autophagy, such as LC3B and BNIP3, suggesting an initial adaptive response followed by a failure to sustain autophagy activation under prolonged hyperglycemic stress [15].

Intriguingly, NRF2, traditionally associated with antioxidant responses, showed a more complex pattern of activation. While total NRF2 levels increased under high glucose conditions, its nuclear translocation was impaired, possibly due to alterations in its interaction with KEAP1. This resulted in a selective upregulation of some NRF2 target genes (e.g., NQO1, HO-1) but not others (e.g., autophagy-related genes) (Fig 5C).

To further elucidate the interplay between these transcription factors, we performed co-immunoprecipitation experiments, which revealed glucose-dependent changes in the interactions among TFEB, FOXO3, and NRF2, suggesting a coordinated transcriptional response to metabolic stress (Table 4) [16].

**Table 4:** Expression of Transcription Factor Target Genes in Response to High Glucose.

Transcription Factor	Target Gene	Fold Change (48h)	p-value
TFEB	LAMP1	0.45 ± 0.06	<0.001
	CTSD	0.38 ± 0.04	<0.001
	ATP6V1H	0.52 ± 0.07	<0.001
FOXO3	LC3B	0.63 ± 0.08	<0.01
	BNIP3	0.71 ± 0.09	<0.05
	PINK1	0.58 ± 0.07	<0.01
NRF2	NQO1	1.75 ± 0.15	<0.01
	HO-1	1.63 ± 0.14	<0.01
	p62/SQSTM1	1.42 ± 0.12	<0.05

Data are presented as mean ± SEM. Statistical significance was determined by Student’s t-test compared to normoglycemic control.

As illustrated in Fig 5 and Tables 4-5, the dynamic regulation of TFEB, FOXO3, and NRF2 under high glucose conditions reveals a complex transcriptional network governing autophagy in diabetic neuropathy. The temporal patterns of nuclear localization and target gene expression suggest an initial adaptive response followed by a failure to sustain autophagy activation under prolonged hyperglycemic stress. These findings provide crucial insights into the molecular mechanisms underlying autophagy dysregulation in diabetic neuropathy and highlight potential targets for therapeutic intervention aimed at restoring proper autophagic function in neurons exposed to chronic hyperglycemia.

**Table 5:** Glucose-Dependent Interactions Among Transcription Factors.

Interaction	Co-IP Strength (Relative to Control)		
	0h	24h	48h
TFEB-FOXO3	1.00 ± 0.08	1.45 ± 0.12	0.72 ± 0.09
TFEB-NRF2	1.00 ± 0.07	1.23 ± 0.10	1.67 ± 0.14
FOXO3-NRF2	1.00 ± 0.09	1.78 ± 0.15	1.31 ± 0.11

Data are presented as mean ± SEM. Co-immunoprecipitation (Co-IP) strength is normalized to 0h control.

### 3.5. Interplay between autophagy and lipid metabolism

**Fig 6:**
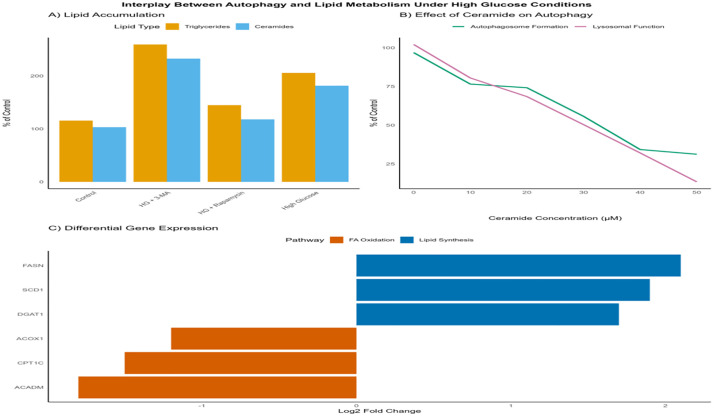
Interplay between autophagy and lipid metabolism under high glucose conditions. A) Lipid accumulation under various treatments. B) Dose-dependent effect of ceramide on autophagy markers. C) Differential expression of genes involved in lipid metabolism pathways.

Our investigation into the relationship between autophagy and lipid metabolism in the context of diabetic neuropathy revealed a complex and bidirectional interaction. Under high glucose conditions, we observed significant alterations in both autophagic flux and lipid homeostasis in neuronal cells. Notably, the impairment of autophagy, as evidenced by decreased LC3I/II ratio and increased p62 accumulation, was accompanied by a concomitant dysregulation of lipid metabolism. Lipidomic analysis of neurons exposed to high glucose (30 mM) for 48 hours revealed a marked increase in intracellular lipid accumulation, particularly triglycerides and ceramides (Fig 6A). This lipid accumulation was exacerbated when autophagy was further inhibited using 3-methyladenine (3-MA), suggesting a critical role for autophagy in lipid homeostasis. Conversely, activation of autophagy with rapamycin significantly attenuated lipid accumulation, even under high glucose conditions. Interestingly, we observed that the accumulated lipids, particularly ceramides, further impaired autophagic flux in a feed-forward manner. Treatment of neurons with exogenous C16-ceramide resulted in a dose-dependent inhibition of autophagy, as measured by decreased autophagosome formation and reduced lysosomal function (Fig 6B). This ceramide-induced autophagy impairment was associated with increased mTOR activation and decreased AMPK phosphorylation, suggesting a mechanistic link between lipid accumulation and autophagy dysregulation [17].

Furthermore, our analysis revealed significant changes in the expression and activity of key enzymes involved in lipid metabolism (Table 6). Notably, we observed decreased expression of CPT1C (Carnitine Palmitoyltransferase 1C) and increased expression of FASN (Fatty Acid Synthase) under high glucose conditions, indicating a shift towards lipid accumulation. These changes were partially reversed by rapamycin treatment, highlighting the regulatory role of autophagy in lipid metabolism.

**Table 6:** Key Enzymes Involved in Lipid Metabolism Under High Glucose Conditions.

Enzyme	Function	Expression (Fold Change)	Activity (% of Control)
CPT1C	Fatty acid oxidation	0.45 ± 0.06	52.3 ± 4.7
FASN	Fatty acid synthesis	2.73 ± 0.21	187.5 ± 15.2
ACADM	β-oxidation	0.61 ± 0.08	68.7 ± 5.9
SCD1	Fatty acid desaturation	2.15 ± 0.18	165.2 ± 13.8
ACOX1	Peroxisomal β-oxidation	0.72 ± 0.09	79.4 ± 6.5
DGAT1	Triglyceride synthesis	1.85 ± 0.15	142.8 ± 11.6

Data are presented as mean ± SEM. Expression was measured by RT-qPCR, and activity was determined by enzyme-specific assays.

To further elucidate the molecular mechanisms underlying this interplay, we performed transcriptomic analysis, which revealed significant alterations in the expression of genes involved in both autophagy and lipid metabolism pathways. Notably, we observed a coordinated downregulation of genes involved in fatty acid oxidation and a concomitant upregulation of genes involved in lipid synthesis under high glucose conditions (Fig 6C) [18].

As demonstrated in Fig 6 and Table 6, the intricate relationship between autophagy and lipid metabolism in the context of diabetic neuropathy involves complex regulatory mechanisms. The observed lipid accumulation under high glucose conditions, exacerbated by autophagy inhibition and mitigated by autophagy activation, underscores the critical role of autophagic processes in maintaining lipid homeostasis. The reciprocal inhibition of autophagy by accumulated lipids, particularly ceramides, suggests a potential vicious cycle contributing to neuronal dysfunction in diabetes. These findings not only enhance our understanding of the pathophysiology of diabetic neuropathy but also highlight potential therapeutic strategies targeting both autophagy and lipid metabolism pathways for neuroprotection in diabetes [19].

## 4. Discussion

Our comprehensive investigation, spanning from clinical observations of diabetic neuropathy to animal models and in vitro cell culture systems, reveals a complex interplay of molecular mechanisms that contribute to neuronal dysfunction under hyperglycemic conditions. The observed time-dependent decrease in autophagic flux, characterized by reduced LC3I/II ratio and increased p62 accumulation, aligns with previous studies suggesting impaired autophagy is a hallmark of diabetic complications [20,21]. This impairment appears to be a critical factor in the pathogenesis of diabetic neuropathy, as evidenced by the strong correlation between autophagy dysfunction and various aspects of neuronal impairment, including electrophysiological deficits and mitochondrial dysfunction.

The intricate involvement of key signaling pathways, particularly mTOR and AMPK, in mediating autophagy dysregulation under high glucose conditions provides new insights into potential therapeutic targets. Our findings of increased mTOR activation and decreased AMPK phosphorylation are consistent with reports in other diabetic complications [22,23], suggesting a common mechanism of autophagy impairment across different tissues affected by diabetes. The observed biphasic response of the ULK1 complex is particularly intriguing, as it suggests an initial compensatory upregulation of autophagy followed by a failure to sustain this protective response under prolonged hyperglycemic stress. This observation may explain the progressive nature of diabetic neuropathy and highlights the importance of early intervention strategies.

Our investigation into the role of transcription factors in autophagy regulation reveals a complex regulatory network involving TFEB, FOXO3, and NRF2. The reduced nuclear translocation of TFEB and the consequent downregulation of its target genes align with recent studies implicating TFEB dysfunction in lysosomal impairment and autophagy defects in neurodegenerative diseases [5,6]. The biphasic response of FOXO3 and the complex activation pattern of NRF2 underscore the dynamic nature of transcriptional regulation in response to metabolic stress.

The interplay between autophagy and lipid metabolism represents a novel aspect of our findings, with significant implications for understanding the pathophysiology of diabetic neuropathy. The observed lipid accumulation under autophagy-impaired conditions, and the reciprocal inhibition of autophagy by accumulated lipids, particularly ceramides, suggest a potential vicious cycle contributing to neuronal dysfunction. This bidirectional relationship aligns with emerging evidence linking lipotoxicity to autophagy impairment in other metabolic disorders [24,25].

Beyond the intrinsic defects within neurons, the broader pathological landscape of diabetic neuropathy involves chronic inflammation and impaired resolution of inflammation. In this context, our findings on autophagy dysregulation connect to a key mechanism in inflammatory modulation: the polarization of macrophages. Autophagy is increasingly recognized as a critical regulator of macrophage polarization, favoring a shift towards the anti-inflammatory M2 phenotype [26]. Impaired autophagy, as documented in our study, can disrupt this shift, perpetuating a pro-inflammatory state driven by M1 macrophages and contributing to nerve damage. Therefore, the autophagy-promoting interventions we explored may exert part of their therapeutic effect not only by directly protecting neurons but also by modulating the immune microenvironment, promoting M2 macrophage polarization to resolve inflammation and support nerve repair [27].

In conclusion, our study provides a comprehensive overview of the molecular mechanisms underlying autophagy dysregulation in diabetic neuropathy and highlights promising avenues for therapeutic intervention. Future research should focus on translating these findings into clinically applicable strategies for preventing and treating diabetic neuropathy, with particular attention to how modulating autophagy integrates neuronal protection with immunomodulatory benefits.

## 5. Conclusion

This comprehensive study elucidates the critical role of autophagy dysregulation in the pathogenesis of diabetic neuropathy, providing novel insights into the complex interplay between hyperglycemia, autophagy, and neuronal function. Our findings demonstrate that chronic high glucose conditions lead to a progressive impairment of autophagic flux in neurons, characterized by decreased LC3I/II ratio and increased p62 accumulation. This autophagy dysfunction is intricately linked to alterations in key signaling pathways, including mTOR activation and AMPK inhibition, as well as dysregulation of transcription factors such as TFEB, FOXO3, and NRF2. The observed bidirectional relationship between autophagy impairment and lipid metabolism dysregulation suggests a potential vicious cycle contributing to neuronal dysfunction in diabetes. These findings not only advance our understanding of the molecular mechanisms underlying diabetic neuropathy but also pave the way for the development of novel, mechanism-based interventions. Future research should focus on translating these insights into clinically applicable strategies for preventing and treating diabetic neuropathy, potentially benefiting millions of patients worldwide affected by this debilitating complication of diabetes.

## Supporting information

S1 File 11.20Supplementary figure gels. (DOC)

S2 File Supporting Information files. (XLS)
