# Supplementary figures and images for: Molecular mechanisms of autophagy disorder in diabetic neuropathy: Focusing on signaling pathways and regulation of lipid metabolism
Source: PLoS One. 2026 Jul 9;21(7):e0344082. doi: 10.1371/journal.pone.0344082 (PMC13349130; doi:10.1371/journal.pone.0344082)

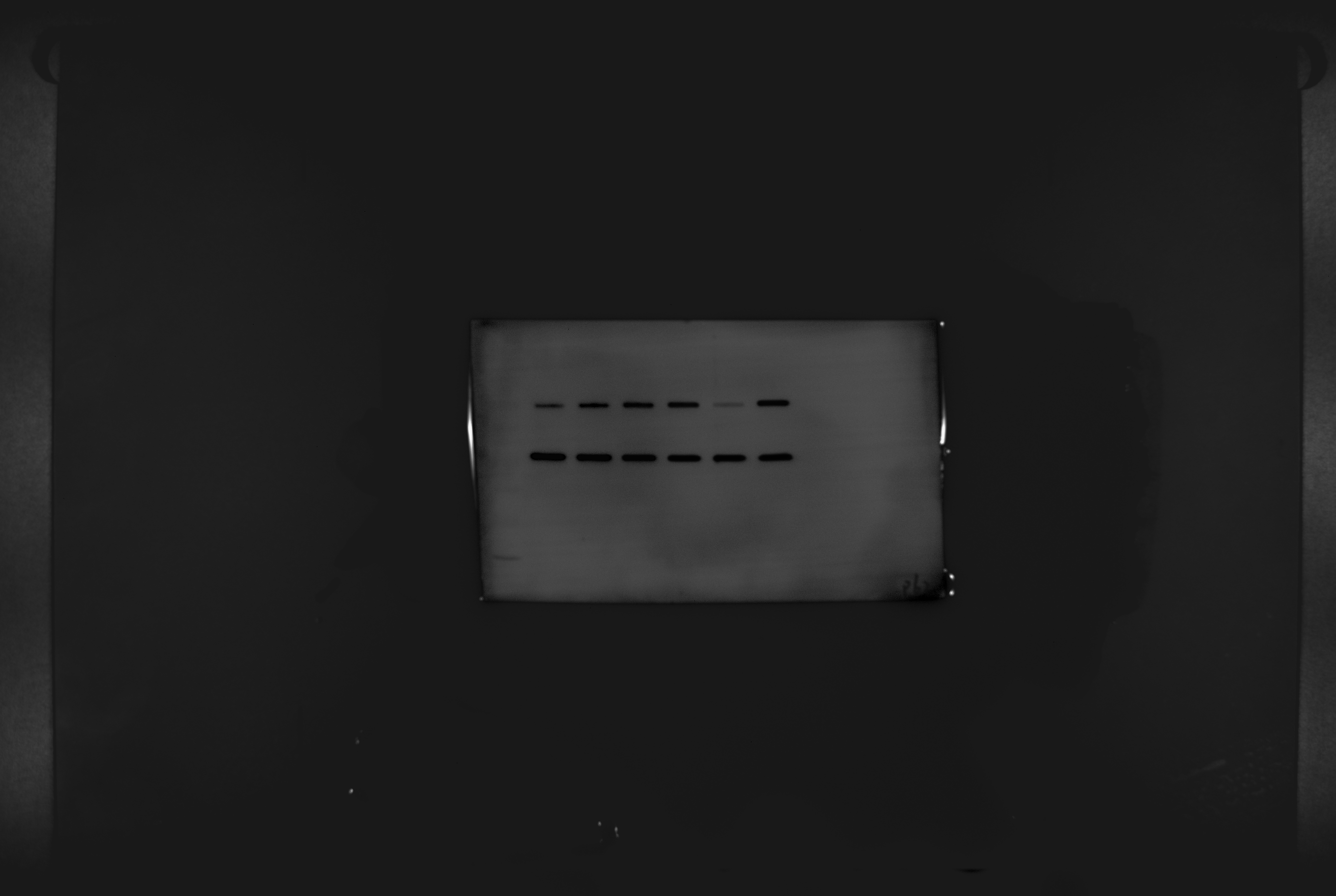

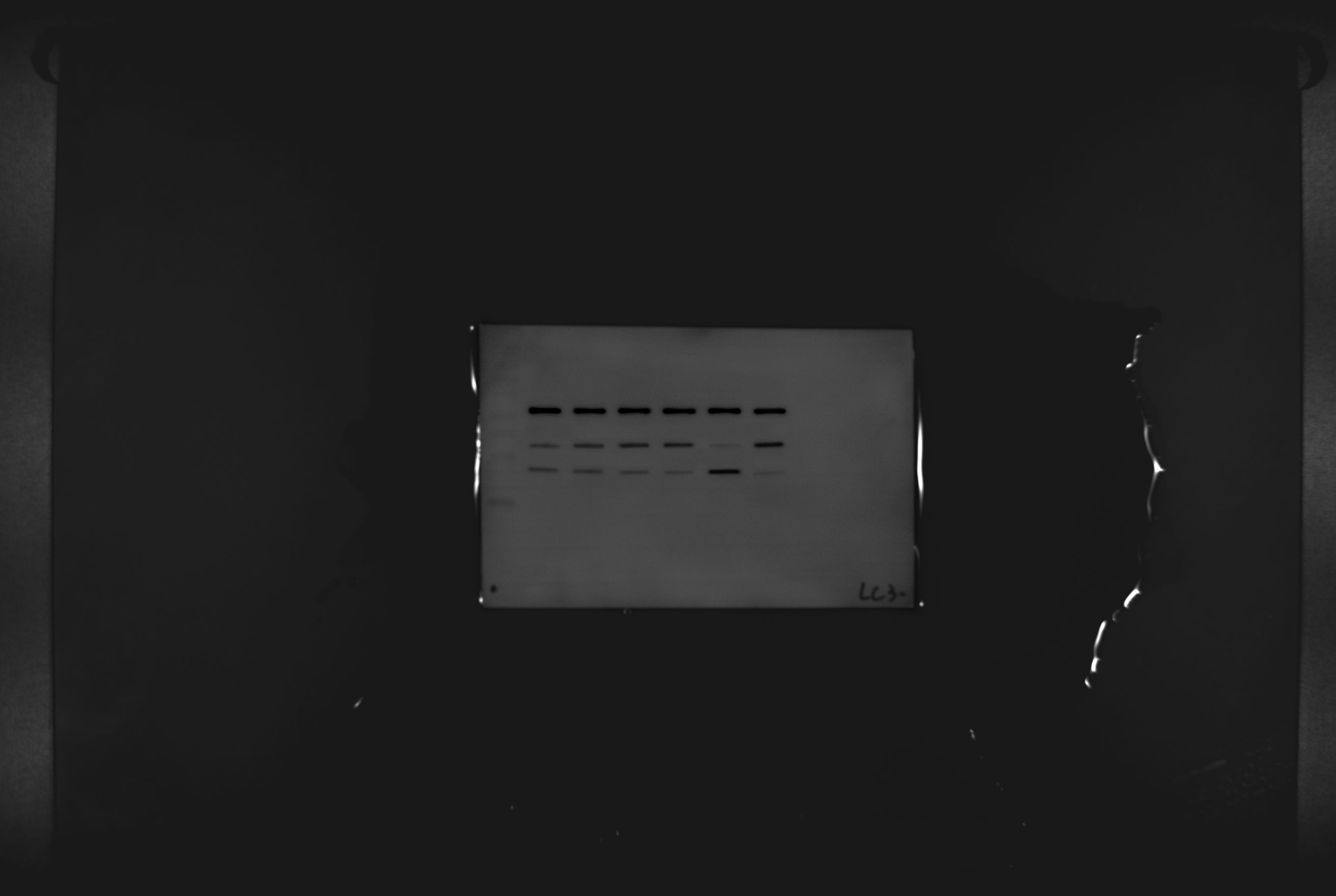


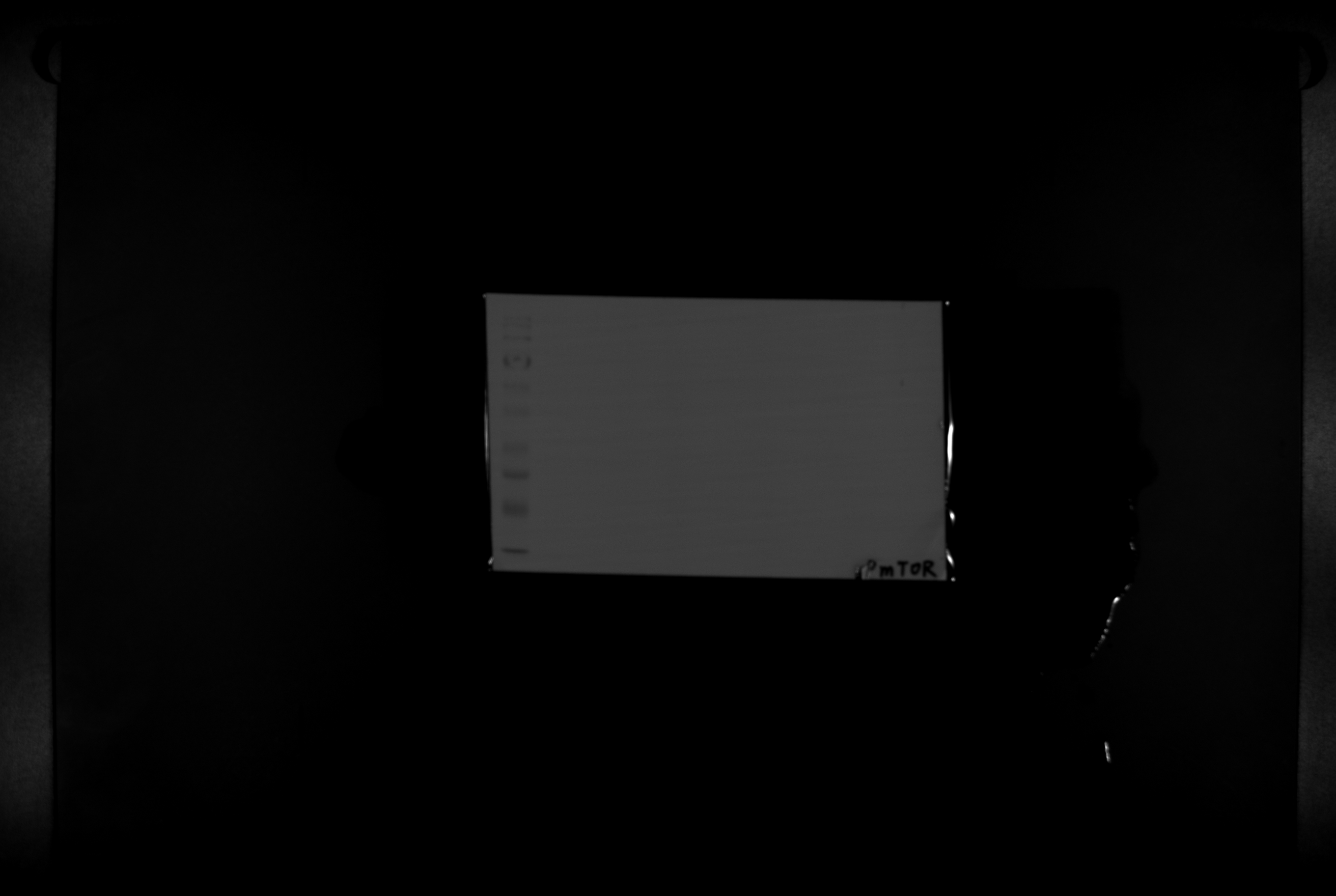


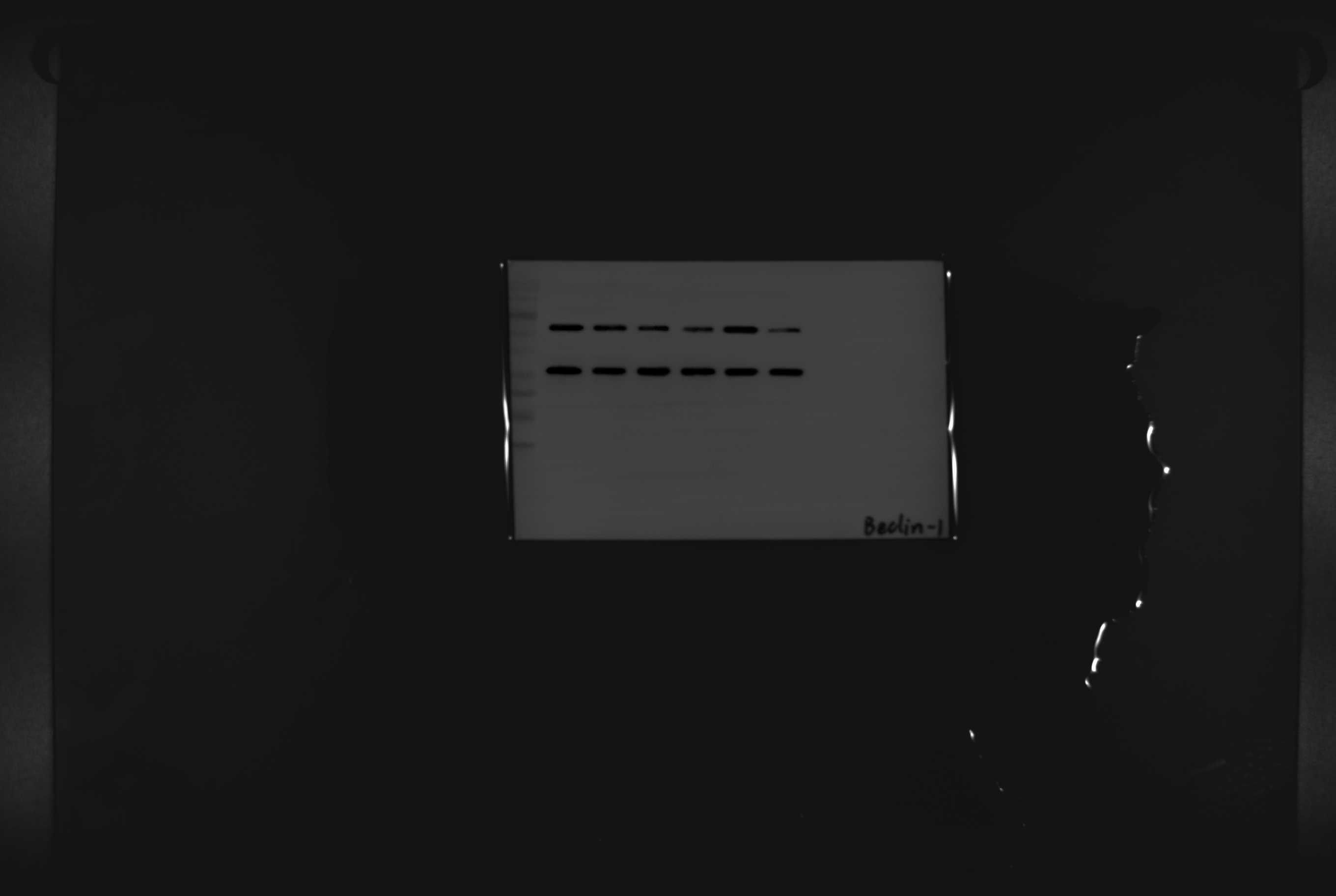


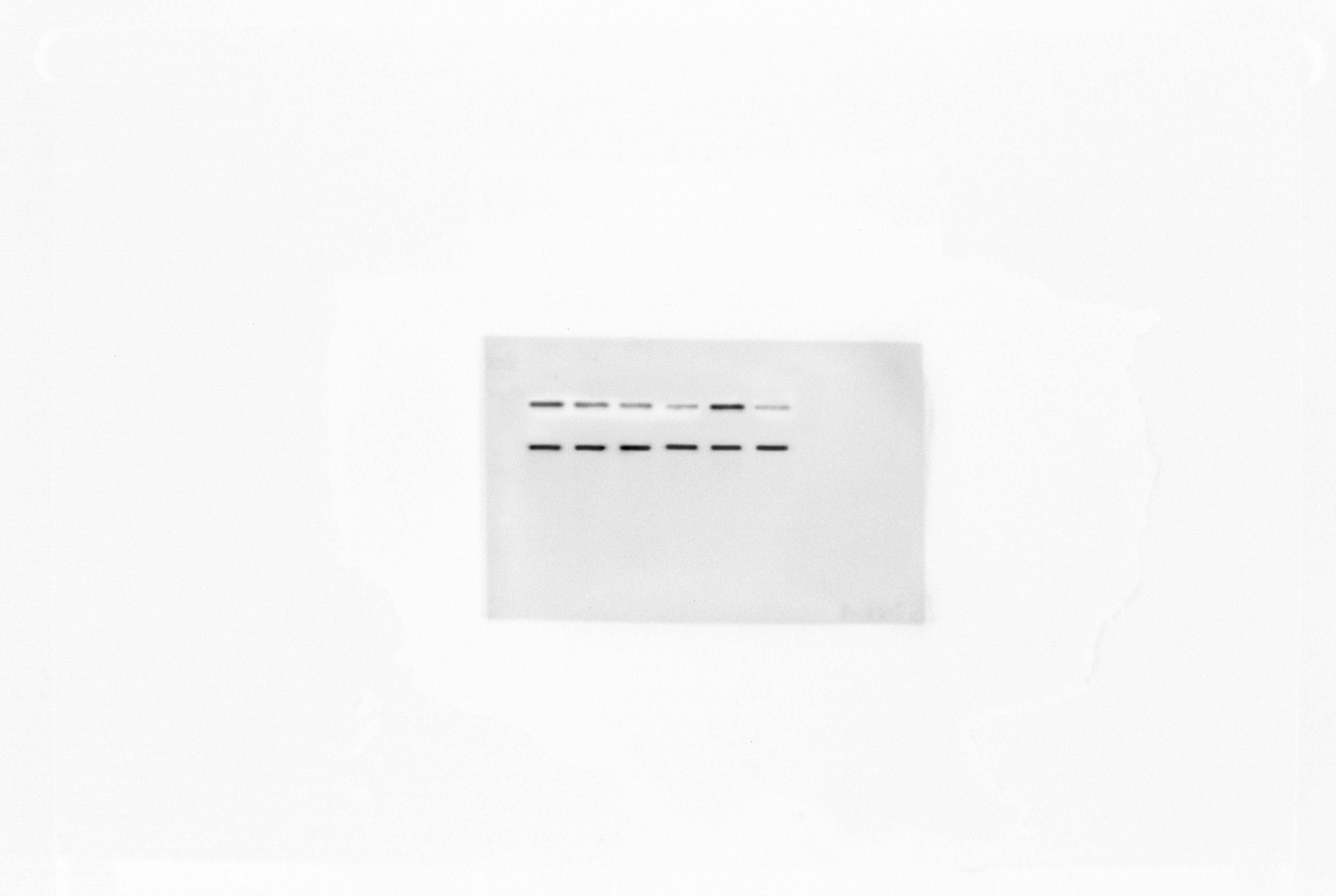


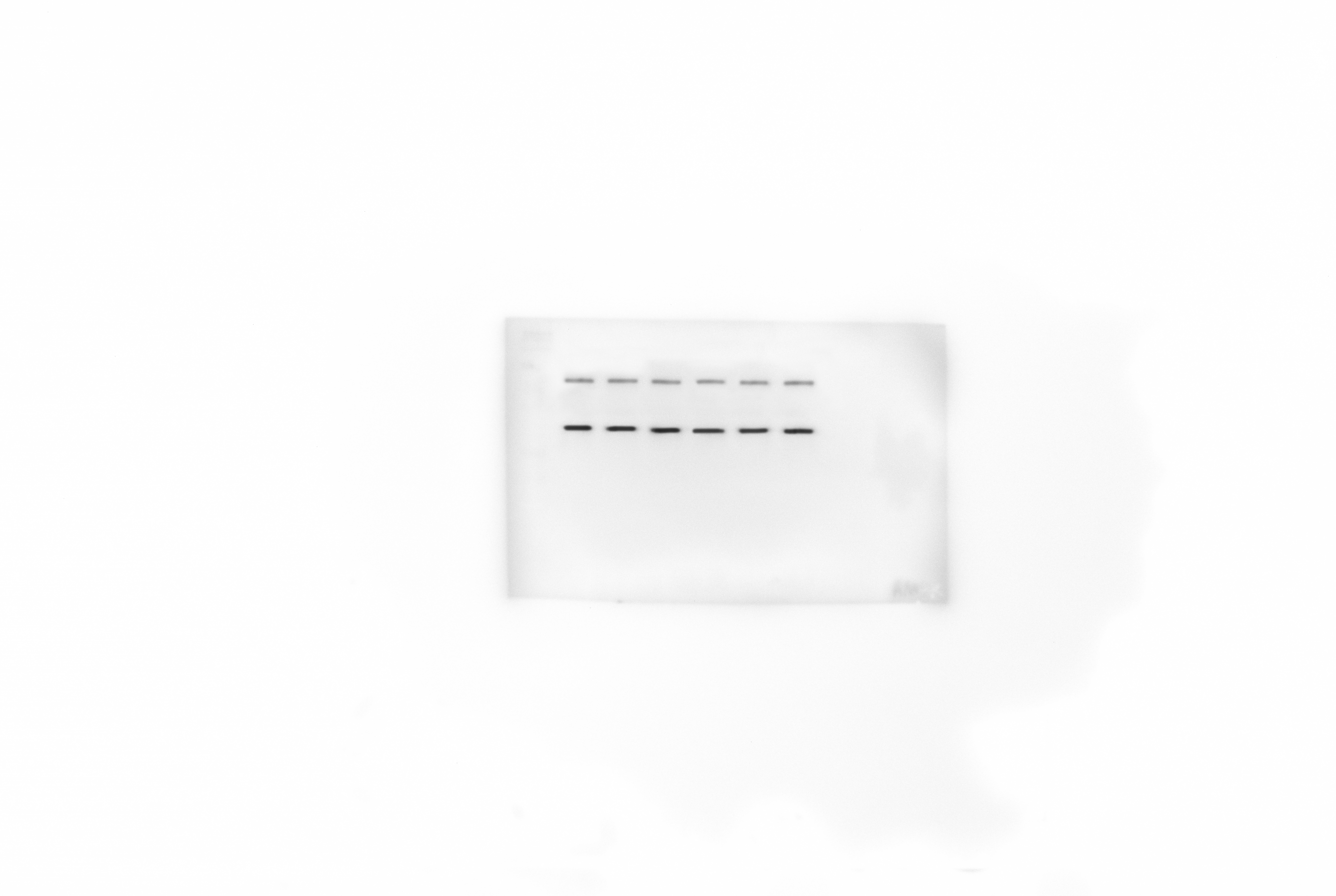


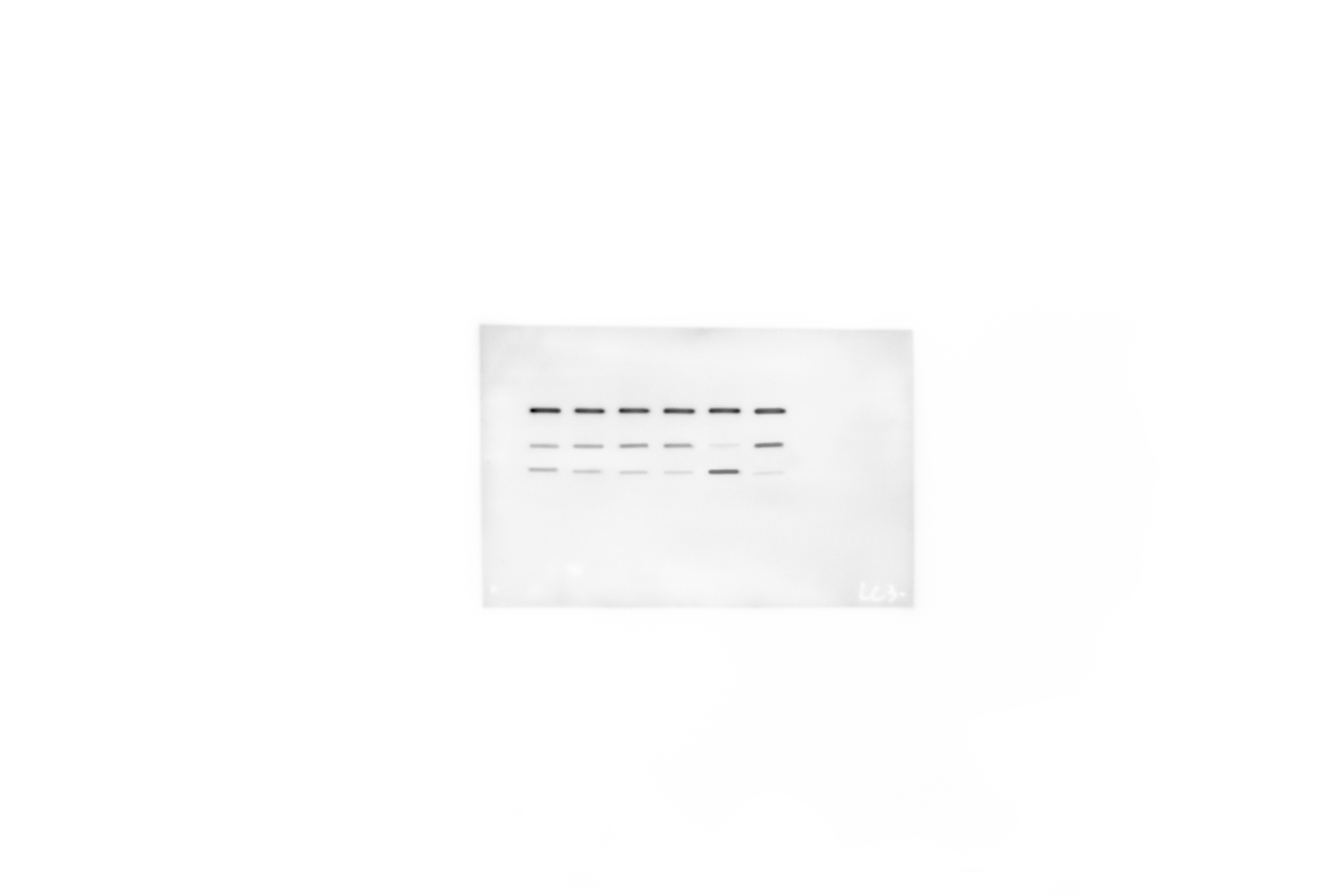


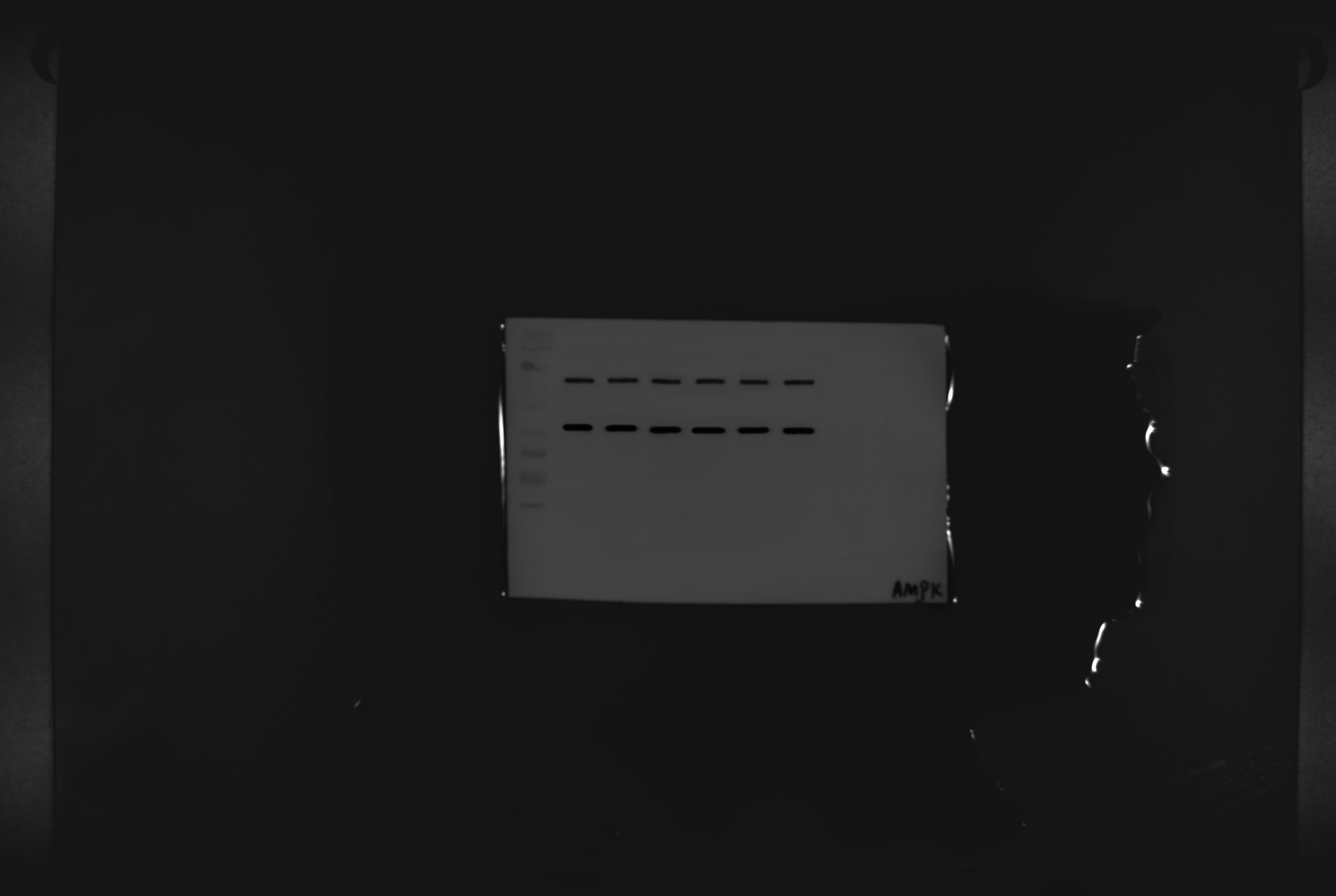


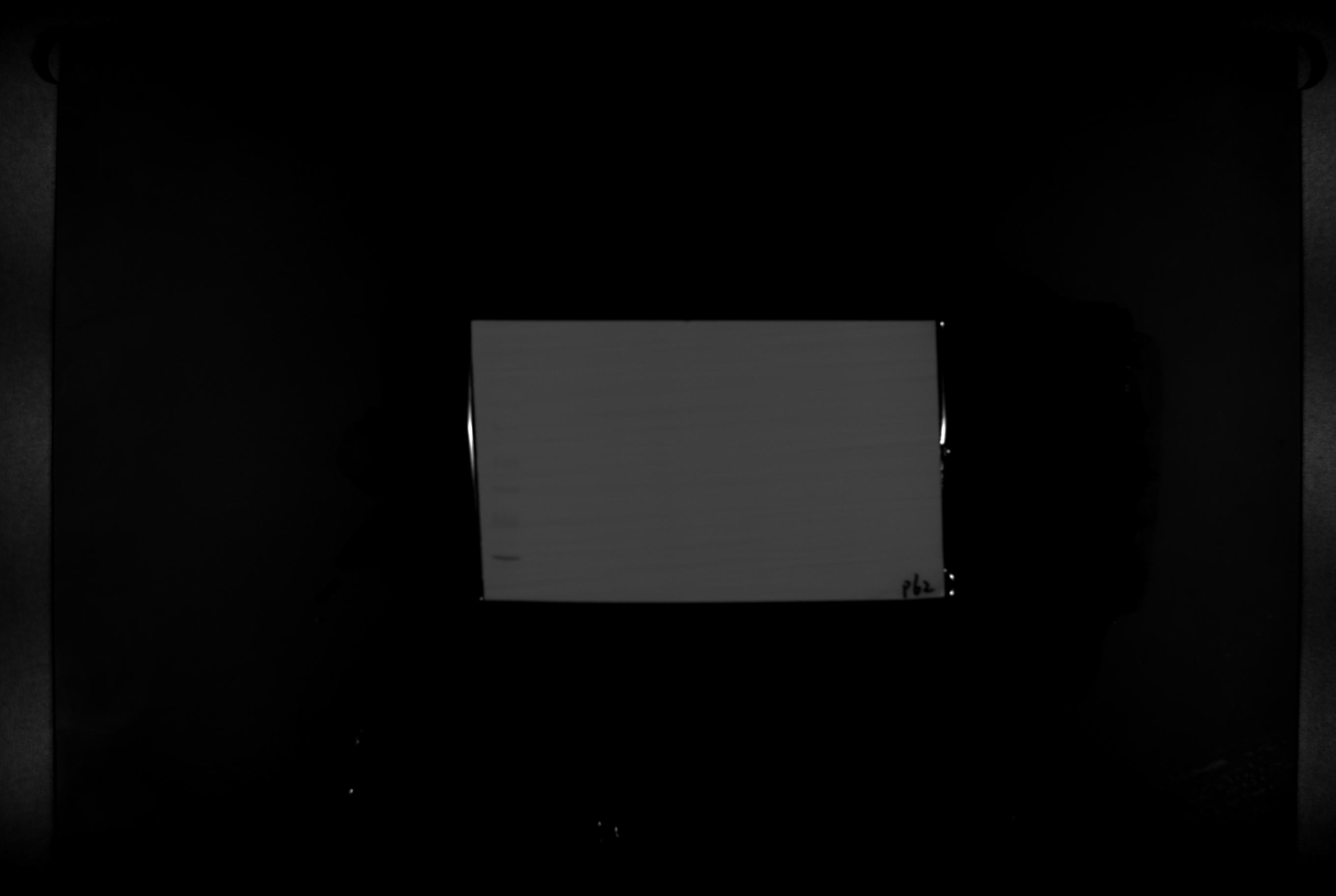


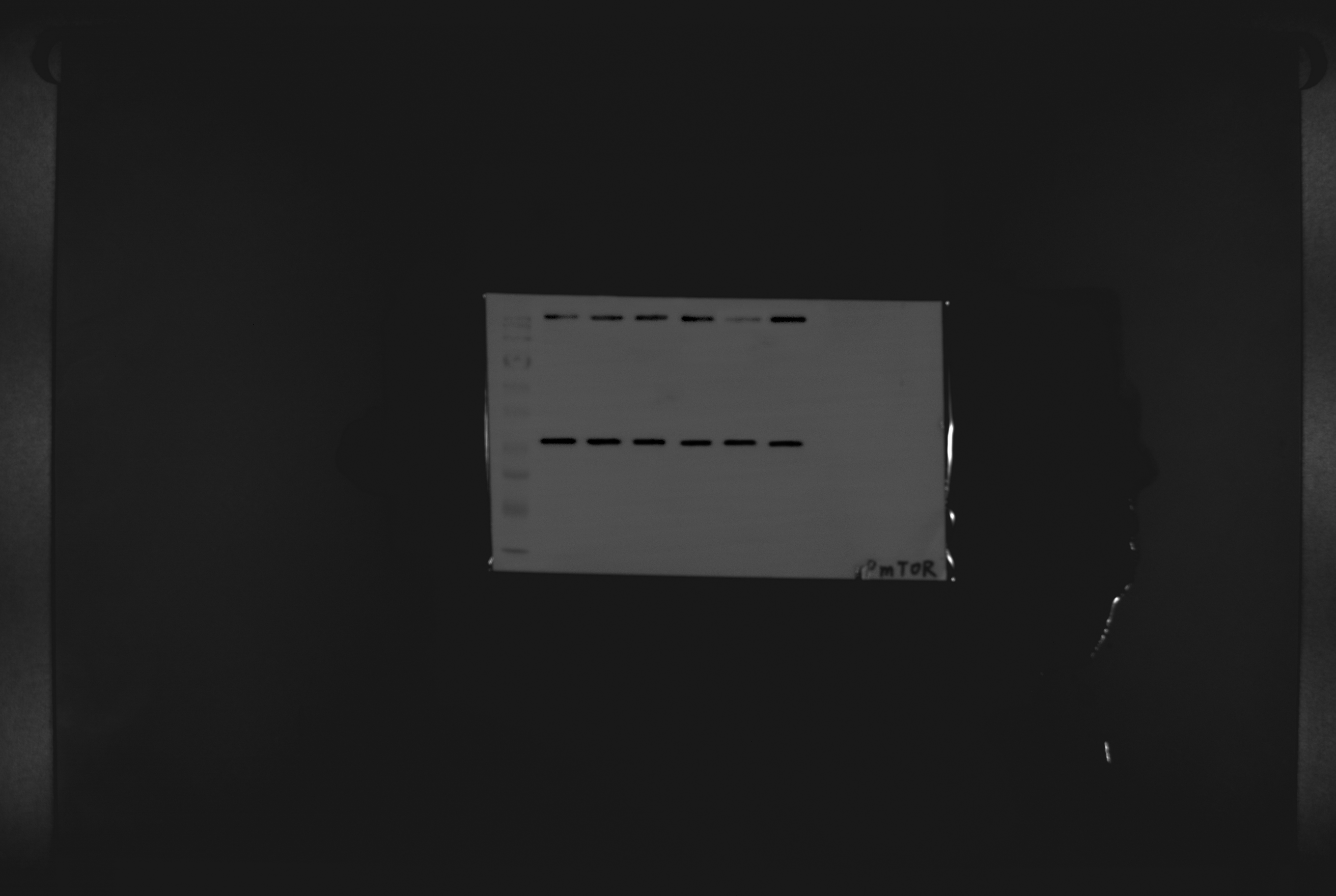

Supplement: S1 File — (DOC) [file pone.0344082.s001.doc]
